# Time-Varying Networks of Inter-Ictal Discharging Reveal Epileptogenic Zone

**DOI:** 10.3389/fncom.2017.00077

**Published:** 2017-08-18

**Authors:** Luyan Zhang, Yi Liang, Fali Li, Hongbin Sun, Wenjing Peng, Peishan Du, Yajing Si, Limeng Song, Liang Yu, Peng Xu

**Affiliations:** ^1^Key Laboratory for NeuroInformation of Ministry of Education, School of Life Science and Technology, University of Electronic Science and Technology of China Chengdu, China; ^2^Department of Neurology, Sichuan Academy of Medical Sciences and Sichuan Provincial People's Hospital Chengdu, China; ^3^Department of Neurology, Affiliated Hospital of University of Electronic Science and Technology of China Chengdu, China; ^4^Center for Information in BioMedicine, University of Electronic Science and Technology of China Chengdu, China

**Keywords:** epilepsy, inter-ictal discharge, adaptive directed transfer function, electroencephalogram, dynamic networks, epileptogenic zone

## Abstract

The neuronal synchronous discharging may cause an epileptic seizure. Currently, most of the studies conducted to investigate the mechanism of epilepsy are based on EEGs or functional magnetic resonance imaging (fMRI) recorded during the ictal discharging or the resting-state, and few studies have probed into the dynamic patterns during the inter-ictal discharging that are much easier to record in clinical applications. Here, we propose a time-varying network analysis based on adaptive directed transfer function to uncover the dynamic brain network patterns during the inter-ictal discharging. In addition, an algorithm based on the time-varying outflow of information derived from the network analysis is developed to detect the epileptogenic zone. The analysis performed revealed the time-varying network patterns during different stages of inter-ictal discharging; the epileptogenic zone was activated prior to the discharge onset then worked as the source to propagate the activity to other brain regions. Consistence between the epileptogenic zones detected by our proposed approach and the actual epileptogenic zones proved that time-varying network analysis could not only reveal the underlying neural mechanism of epilepsy, but also function as a useful tool in detecting the epileptogenic zone based on the EEGs in the inter-ictal discharging.

## Introduction

Being a brain disease rather than a disorder, the neuronal synchronous discharging may cause an epileptic seizure (Fisher et al., 2014). Epileptic seizure is always accompanied by various clinical manifestations, such as loss of consciousness, movement dysfunction, and etc. (Cheung et al., 2006; Hommet et al., 2006; Malfait and Lippé, 2011; Blumenfeld, 2012). One disturbing issue is that the epileptic seizure is unpredictable, which imposes both the epilepsy patients (EPs) and their families with great burden, such as difficulty in seeking jobs, financial strain, and low quality of life (Canuet et al., 2009; Shanmukhi et al., 2015); thus the need for more effective therapies of epilepsy. Currently, the clinical therapies including taking drugs and surgery have been widely considered to control epileptic seizure (Huang et al., 2017; Mula, 2017). In clinical therapy, doctors always ask the patients to first try the antiepileptic drugs and later surgery is usually considered for the patients when the drugs become ineffective. Therefore, finding the accurate location of the epileptogenic zone is of great importance in the clinical therapy of epilepsy. Generally, epileptogenic zone is defined as the area that accounts for the generation of clinical seizures in the cerebral cortex (Nadler and Spencer, 2014).

Some previous studies already investigated the epileptogenic zone during both ictal and postictal discharging (Gersch and Goddard, 1970; Spanaki et al., 1998, 1999; Avery et al., 1999). For a example, study based on the single-photon emission computer tomography (SPECT) reported that during ictal discharging, the epileptogenic zone showed an increased perfusion (Spanaki et al., 1999). Moreover, another study based on the inter-ictal and ictal discharging has also compared the performance of various non-invasive electrophysiological and imaging techniques (Stefan et al., 1987), and found that PET showed focal abnormalities in all cases, while electroencephalogram (EEG) and magnetic resonance imaging (MRI) revealed the focal abnormalities in most cases. Long-term EEG recording plays an important role in non-invasive presurgical (level I) diagnosis, and compared with the routine EEG examinations, it allows the assessment of constancy and time sequence of the focal ictal onset and its propagation. In addition, MRI provides an excellent anatomical presentation of structural abnormalities with relatively high sensitivity, while PET-CT has the highest sensitivity in localizing the epileptogenic zone compared with MRI and EEG. This provides reliable information on both anatomical localization and extent of functional abnormalities in various brain regions (Shao et al., 2014).

Among these techniques, long-term EEG recording show obvious advantages, such as the high temporal resolution, low cost, and easy availability. Recently, many studies have reported some interesting findings based on the intracranial ictal EEGs (Kramer et al., 2008; Wilke et al., 2010). For example, the activated brain regions in gamma band during the ictal discharging showed the greatest overlap with the seizure onset foci determined by epileptologists (Wilke et al., 2011), and research based on the ictal intracranial EEG of patients with bilaterally synchronous epileptiform discharging implied that brain regions with high outflow corresponded to the surgical resection regions (Cho et al., 2011). Comparing to intracranial EEG, the scalp EEG is much more acceptable for the EPs, as the intracranial recording causes damages to their brains. In reference to the localization of epileptogenic zone, a study based on the scalp EEG found that the location of source image showed high correlation with the brain region that had been resected surgically (Lu et al., 2012).

The afore-mentioned studies are mainly based on the EEG recorded during the ictal discharging, however, the problem is that ictal discharging is not so easy to be captured. In contrast, the inter-ictal discharging is relatively easily to be recorded during the 24-h video-EEG monitoring. Previous studies showed that the specific regional activity of the ictal discharging could be observed during the inter-ictal discharging (Stefan et al., 1987; Wilke et al., 2011); in other words, the activated brain regions during inter-ictal discharging period showed the high similarity with that during the epileptic seizure. Thereby, the inter-ictal discharging can also be used to perform similar analysis as those conducted during the ictal discharging (Hufnagel et al., 2000; Mormann et al., 2000; Asano et al., 2003; Marsh et al., 2010; Wilke et al., 2011; Diessen et al., 2013; Song et al., 2013; Shao et al., 2014). For example, Wilke et al. performed a source localization on four inter-ictal discharging from one EP, and found that the identified sources lied within or surrounding areas of one of the foci (Wilke et al., 2008). Furthermore, they compared the activated areas in ictal, inter-ictal, and inter-ictal spikes periods, and found that the location of the activated gamma networks identified during inter-ictal spikes were similar to that of ictal gamma networks which has the smallest distance from seizure onset zone (Wilke et al., 2011).

There are several methods utilized in epilepsy research such as mutual information (Mars and Lopes da Silva, 1987), Granger causality analysis (Liao et al., 2010; Epstein et al., 2014), among others. Epstein et al. performed Granger causality analysis on intracranial EEG to analyze the features of preictal networks, and found that the Granger causality network analysis may aid surgical outcome in cases of ambiguous intracranial EEG onset (Epstein et al., 2014). Jiao et al. employed Granger causality to investigate the role of medial temporal lobe in epilepsy (mTLE), and found that the seizure is propagated from the medial temporal lobe to other regions (Jiao et al., 2009). Due to the fact that epilepsy seizure and inter-ictal discharging are caused by neural abnormal discharging (Staley et al., 2005), although Granger causality analysis can provide some information about epileptic mechanism in a time window, it cannot reflect the brain network changes with the ongoing time. It is well-known that the information can be efficiently processed in tens of milliseconds in human brain. As for epilepsy, a gradual evolution of brain networks actually exists during abnormal inter-ictal discharging, i.e., different network patterns may be revealed in the different stages of inter-ictal discharging. Therefore, we assume that the time-varying network patterns may provide useful information to uncover the abnormal information processing and propagation when inter-ictal discharging is observed. Consequently, the time-varying network analysis (i.e., adaptive directed transfer function) which can be applied to investigate the dynamic network patterns during certain task (Li et al., 2016) is vital in establishing the corresponding time-varying networks during inter-ictal discharging. Moreover, given the fact that the inter-ictal spiking activity presented here is similar to ictal activity (Wilke et al., 2011) and the inter-ictal spiking yet easier to be obtained than ictal data. This shows that probing the mechanism of inter-ictal discharging is very important. Therefore, in this study the time-varying analysis was applied to investigate the dynamic network patterns of EPs based on the inter-ictal discharging, and also to probe the feasibility to locate the corresponding epileptogenic zone using the time-varying network information.

## Materials and methods

### Participants

Three EPs included in this study were diagnosed by the doctors at the Sichuan Academy of Medical Sciences & Sichuan Provincial People's Hospital. This study was carried out in accordance with the recommendations of Medical Ethics Committee of Sichuan Academy of Medical Sciences & Sichuan Provincial People's Hospital with written informed consent from all the subjects. Three subjects with diagnosed epileptogenic zone confirmed by MRI scanning were involved in the experiment. All subjects gave written informed consent in accordance with the Declaration of Helsinki. The protocol was approved by the Medical Ethics Committee of Sichuan Academy of Medical Sciences & Sichuan Provincial People's Hospital. Before the 24-h EEG monitoring, epilepsy patients were required not to take the antiepileptic drugs. The detailed information about these three EPs is shown in Table 1.

**Table 1:** The detailed information about the epilepsy patients.

**Patients**	**EP1**	**EP2**	**EP3**
Age	42	33	36
Gender	Male	Male	Female
Epileptogenic zone	Left temporal lobe	Right amygdala and hippocampus	Left frontal lobe

### Twenty-four hours EEG recording

The EEG datasets (Datasheet in Supplementary Material) of the 16 electrodes (Fp1, Fp2, F3, F4, C3, C4, P3, P4, O1, O2, F7, F8, T3, T4, T5, and T6) derived from the extended international 10–20 system were recorded by using the Australian COMPUMEDICS Greal series of digital video EEG with a sampling rate of 256 Hz. The electrocardiogram and electromyogram were also recorded by the other two extended electrodes. During the 24-h monitoring, a single or dual cameras were synchronously used to monitor the patients' behavior. Moreover, during the 24-h EEG monitoring, the patient would experience at least one complete awake-sleep-wake cycle.

### EEG data analysis

**Figure 1:**
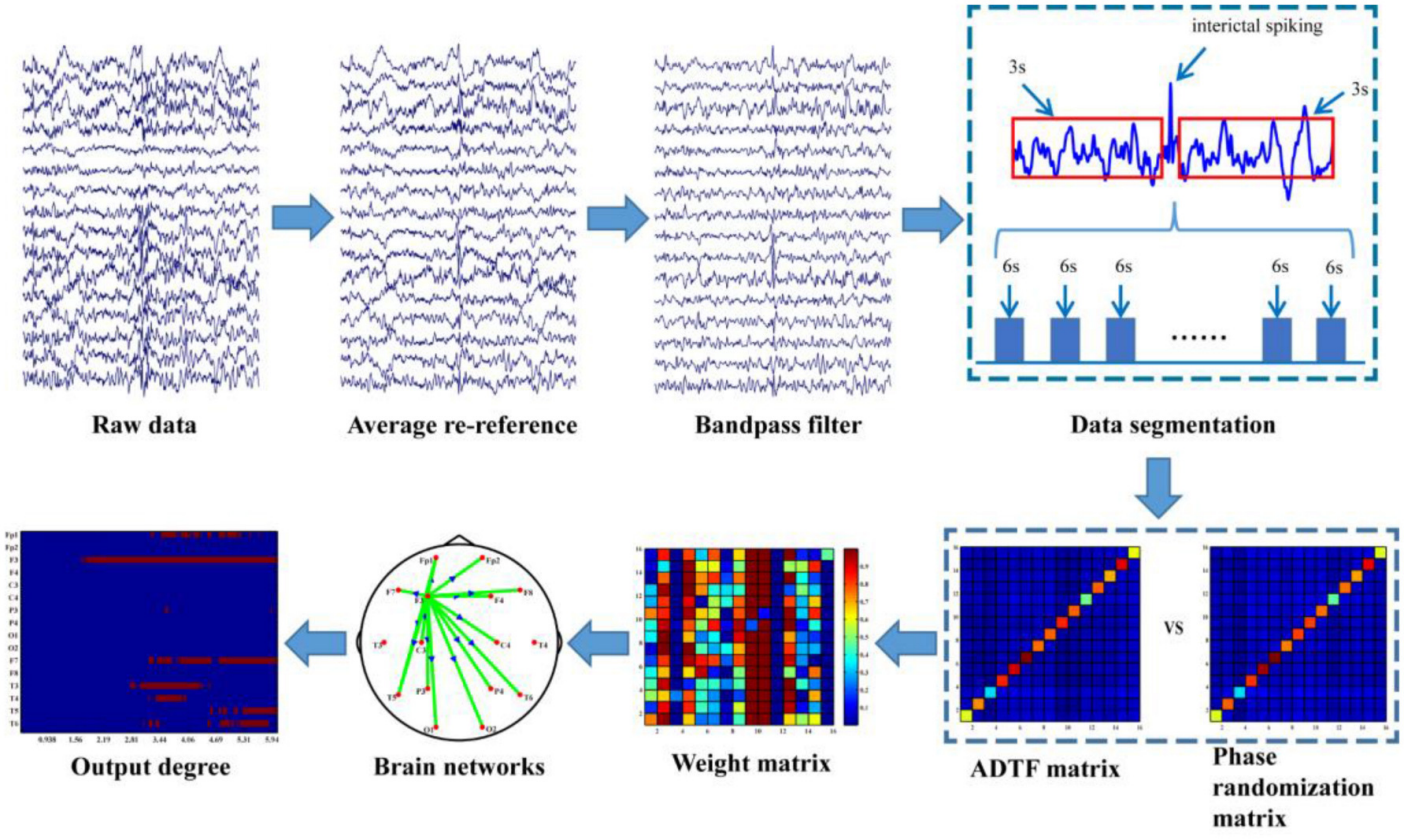
Analysis procedure of inter-ictal discharging.

The data analysis procedure is depicted in Figure 1 and the detailed information about the procedure is further discussed in the following sections.

#### Time-varying network analysis

EEG was pre-processed by averaging, re-referencing and 0.5–30 Hz band-pass filtering prior to the time-varying network analysis. The time-varying network analysis usually requires several segmentations (i.e., trials in evoked EEG experiment) to enable the construction of a reliable network in order to capture the brain architectures and networks (Hu et al., 2012; Li et al., 2016). In this work, compared to the time-varying network analysis for the evoked EEG that usually has the definite stimulus labels, one drawback here is that no exact events were labeled for the inter-ictal discharging during the 24-h EEG monitoring. Hence, labeling inter-ictal discharging is required for constructing the time-varying networks. Due to the fact that there are usually many artifacts during EEG recording and the false recognition rate of the algorithm is high thus in our study the inter-ictal discharging was labeled one by one manually by the epileptologists. For each labeled discharging event the time point corresponding to the peak of inter-ictal discharging is set as time “0,” then 3-s data before “0” and 3-s data after “0” is extracted, which leads to the total 6-s length for each discharging segment. Next, to reduce the calculation load in the following time-varying network analysis, these segments are further 8-rate down-sampled (Li et al., 2016), resulting in 32 Hz (i.e., 192 sample points in the 6-s long segment). In order to uncover the dynamic information processing during inter-ictal discharging, ADTF was used to construct the time-varying networks (Wilke et al., 2007, 2009); and statistical analysis were further utilized to identify the dynamic network patterns during the inter-ictal discharging.

#### Time-varying multivariate adaptive autoregressive (tv-MVAAR) model

For each artifact-free segment, the tv-MVAAR model is defined as

(1) $$\begin{array}{l} X\left(t\right)=\overset{p}{\underset{i=1}{\sum }}A\left(i,t\right)X\left(t-i\right)+E\left(t\right) \end{array}$$

where *X(t)* is the data vector of EEG signal, *E(t)* is the multivariate independent white noise, *X(i,t)* is the matrix of tv-MVAAR model coefficients that is estimated by Kalman filter algorithm (Arnold et al., 1998; Hu et al., 2012), and *p* is order of the model that is automatically determined by the Akaike Information Criterion (AIC) within the range of 2–20 as,

(2) $$\begin{array}{l} AIC\left(p\right)=\ln\left[\det\left(\chi \right)\right]+2M^{2}p/N \end{array}$$

where *M* represents the number of the electrodes, *p* represents the optimal order of the model, *N* represents the number of the time points of each time series and χ represents the corresponding covariance matrix.

#### Adaptive directed transfer function

Parameters *A(f,t)* and *H(f,t)* in the frequency domain are defined as follows;

(3) $$\begin{array}{l} A\left(f,t\right)=\overset{p}{\underset{k=0}{\sum }}A_{k}\left(t\right)e^{-j2\pi f\Delta tk} \end{array}$$

(4) $$\begin{array}{l} A\left(f,t\right)X\left(f,t\right)=E\left(f,t\right) \end{array}$$

(5) $$\begin{array}{l} X\left(f,t\right)=A^{-1}\left(f,t\right)E\left(f,t\right)=H\left(f,t\right)E\left(f,t\right) \end{array}$$

where *A*_*k*_ denotes the matrix of the tv-MVAAR model coefficients, *X(f,t)* and *E(f,t)* are the Fourier transformations of *X(t)* and *X(t)* in the frequency domain, respectively.

Moreover, the normalized ADTF describing the directed flow from the *j*th to the *i*th node is defined in Equation (6), and the final integrated ADTF is defined in Equation (7) within the frequency band of interest (i.e., 0.5–14.5 Hz in this work) as follows;

(6) $$\begin{array}{l} \gamma_{ij}^{2}\left(f,t\right)=\frac{{\left|H_{ij}\left(f,t\right)\right|}^{2}}{\overset{n}{\underset{m=1}{\sum }}{\left|H_{im}\left(f,t\right)\right|}^{2}} \end{array}$$

(7) $$\begin{array}{l} Q_{ij}^{2}\left(t\right)=\frac{\overset{f_{2}}{\underset{k=f_{1}}{\sum }}\gamma_{ij}^{2}\left(k,t\right)}{f_{2}-f_{1}} \end{array}$$

The normalized total information outflow of the *j*th node is further estimated in Equation (8) as;

(8) $$\begin{array}{l} Q_{j}^{2}\left(t\right)=\frac{\overset{n}{\underset{k=1}{\sum }}Q_{kj}^{2}\left(t\right)}{n-1},\;for\;k\neq j \end{array}$$

where *n* is the total number of nodes.

#### Surrogate data

Since ADTF is highly non-linearly correlated with the time series from which it is derived, estimators distribution under the null hypothesis of no connectivity is not well-established. To solve this problem, phase randomization was applied to construct the reference signal (Wilke et al., 2008). Here, Fourier coefficient phases were randomly and independently shuffled to produce the corresponding reference signals considering that phase randomization preserves the spectral structure of the time series. The corresponding reference signal was also used to measure the time-varying connectivity. This procedure was repeated 200 times for each segment of each subject to create an empirical distribution of ADTF values under the null hypothesis of no causal interaction in each edge. The means and variances were then used to calculate the Gauss cumulative distribution. Finally, dynamic networks were calculated with a significance of 0.01 for each EPs.

### Epileptogenic zone localization

Given the fact that the abnormal discharging usually originates from the epileptogenic zone, and gradually propagates to other brain regions, we used the out-degree to measure the origin and the propagation characteristics of inter-ictal discharging. Based on the time-varying networks the out-degree of each node across each sample time point was further calculated by Equation (9) as,

(9) $$\begin{array}{l} k_{i}\left(t\right)=\underset{j\in N}{\sum }a_{ij}\left(t\right),\;for\;i\neq j \end{array}$$

where *N* is the set of all nodes in the network. *a*_*ij*_*(t)* is the connection from node *i* to node *j* at time point *t*, and *a*_*ij*_*(t)* = 1 if the corresponding connection exists, otherwise *a*_*ij*_*(t)* = 0.

After the out-degree of each electrode has been calculated for each sample time point, we can get an out-degree matrix denoting the time-varying out-degree of each node across different time points. The out-degree matrix is further employed to locate the epileptogenic zone for each patient by determining the first appearance of each node compared to the background activity pattern (background means before the epileptogenic zones are activated and it also has some fundamental activities in the brain which maintain normal human activity). For each time point we first identified whether or not this electrode has a weak out-degree (i.e., less than three). If it has a weak out-degree then the corresponding place in the binary out-degree matrix is given a small weight value (i.e., zero); otherwise, a high weight value (i.e., one) is assigned. Based on the binarized out-degree matrix, we can identify the appearance of the earliest electrode that possesses a strong out-degree apart from those electrodes with background outflow (the out-degree pattern at the onset). A threshold of three was applied to remove the interference from the other factors (i.e., noises). Below is a detailed overview of the procedure employed. The corresponding pseudo-codes are included in the appendix.

Step 1. Calculating the connectivity matrix for each patient;
Step 2. Calculating the out-degree matrix. The out-degree matrix comprises of the out-degree of each electrode in the network at each time point;
Step 3. Constructing the binarized out-degree matrix. If the out-degree is more than three the corresponding value of the binarized out-degree matrix is set one, otherwise zero.
Step 4. Constructing the binarized out-degree matrix with the electrode that has the earliest appearance of out-degree as identified apart from the background activity.

The surrounding brain area near the identified electrode is identified as the corresponding epileptogenic zone.

## Results

### Dynamic network patterns

**Figure 2:**
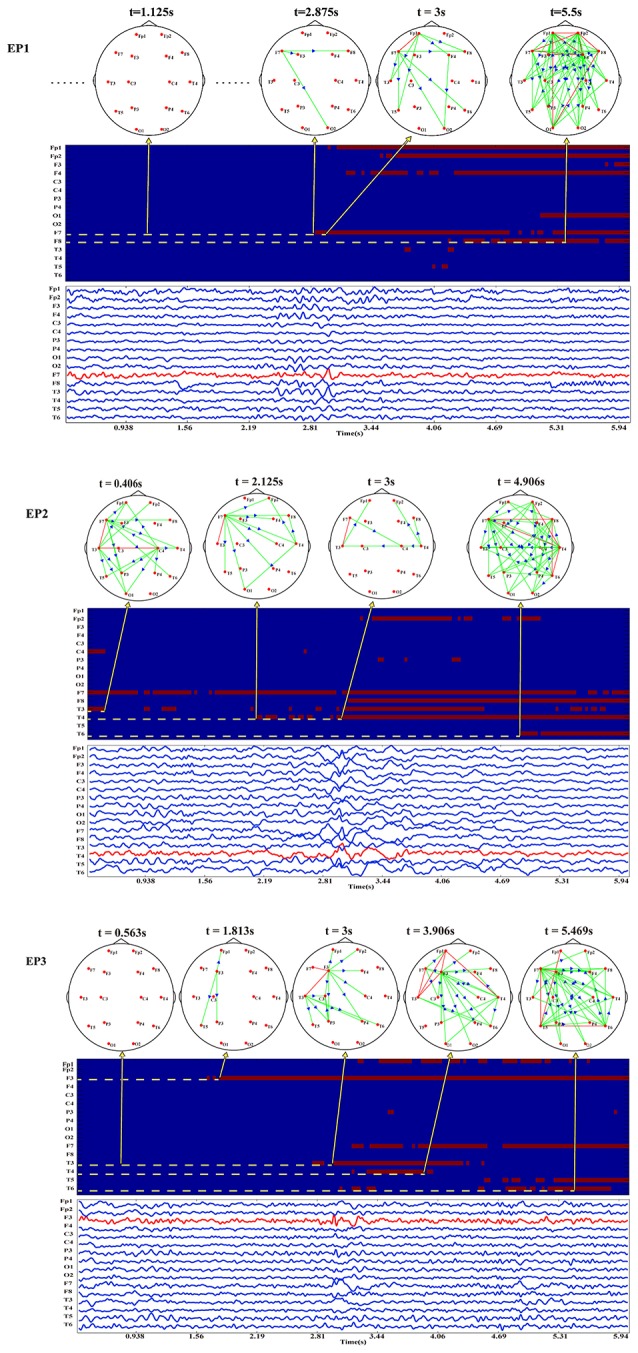
The EEG waveforms, time-varying networks and the binarized time-varying out-degrees for EP1, EP2, and EP3.

Based on the above time-varying network analysis, for each EP, the corresponding time-varying network patterns of the inter-ictal discharging are shown in Figure 2.

It is noteworthy that the EEG waveforms presented are stable at the early stage and approximately at the 3rd second, apart from the electrodes nearby the epileptogenic zones, other electrodes also exhibit the characteristic peaks of inter-ictal discharging, which makes it difficult to localize the epileptogenic zone merely based on EEG waveforms. Notwithstanding, the time-varying network patterns of these Eps from different epileptogenic zones shown in Figure 2 have different network hub nodes location in the brain areas. Moreover, the partial and local brain regions close to the epileptogenic zones are activated at the early stage before the discharging then gradually expand to other brain regions such as the anterior temporal lobe, right temporal lobe, and left frontal lobe. The three Eps show an abnormal inter-ictal discharging at approximately the 3rd second. Compared the network topology before the third second, the networks of the three EPs at the 3rd second actually show much denser patterns. This indicates that more brain areas are involved in the synchronization discharging at this time point.

### Locating epileptogenic zone

Following the procedure for epileptogenic zone localization, we can estimate the threshold-weighted matrices varied across time as shown in Figure 2. Moreover, it is clearly seen that before the actual inter-ictal discharging irrespective of the strong or weak background activities the electrode node close to the epileptogenic zone initially exhibits a strong outflow prior to other electrodes.

Consistently, electrode F7 which is located at the left anterior temporal lobe of EP1 initially firstly appears to have abnormal inter-ictal discharging which then propagate to other brain areas. EP3 also shows a similar outflow pattern with electrode F3 located at the left frontal lobe initially being activated as the crucial sources. As for EP2, besides from the relatively strong background activities, electrode T4 located in the right temporal lobe is observed to be newly activated. Based on the proposed epileptogenic zone localization strategy, the three electrodes (i.e., F7 for EP1, T4 for EP2, and F3 for EP3) close to the epileptogenic zones confirmed by the clinical doctors could be automatically detected based on the time-varying networks.

## Discussion

The abnormal synchronous discharging of the cortical neurons leads to the ictal or inter-ictal discharging (Staley et al., 2005), which can be observed in EPs. Various previous studies have been conducted to investigate the mechanism of epilepsy based on resting-state fMRI and ictal EEG (mainly the intracranial EEG) (Luo et al., 2011; Wang et al., 2011; Zhang et al., 2011; Burns et al., 2013), but few has focused on the dynamic information processing during the inter-ictal discharging, nor the epileptogenic zone localization based on the dynamic network analysis. As the fact that the information can be efficiently processed within tens of milliseconds in human brain, the time-varying network analysis can be used to probe the underlying mechanism of the corresponding dynamic information processing. Then, based on these dynamic networks, we can further probe the feasibility to localize the epileptogenic zone for the EPs. In our present study, 24-h EEG monitoring was performed for all the EPs at the hospital, and this was followed by the application of ADTF and feature discrimination to construct dynamic networks and locate the epileptogenic zone, respectively. In ADTF based time-varying analysis it is necessary to label the inter-ictal discharging for constructing the time-varying networks. To guarantee the labeling accuracy in this study, the inter-ictal discharging is manually labeled by the experienced epileptologists, though this may provide an additional load. In future work, we will develop or seek an algorithm to detect the inter-ictal discharging automatically, and to make it convenient in analysis during the clinical application situations.

The inter-ictal discharging observed in many electrodes as shown in Figure 2 demonstrates the difficulty to localize the epileptogenic zone using the original EEG waveforms. This phenomenon also reveals other aspects of epilepsy, i.e., the abnormal discharging of epilepsy may be propagated from the epileptogenic zone to other brain areas, which forms specific networks for information propagation and processing. Moreover, different time-varying networks for the three EPs having the different epileptogenic zones are actually revealed. By combing the specific epileptogenic zone information, the time-varying networks of the three EPs have a similar evolution pattern. Specifically, at the early stage before the inter-ictal discharging, the brain actually exhibits different background network patterns, where EP1 and EP3 have relatively weaker background networks with smaller outflows, while EP2 shows a relatively stronger background networks with larger outflows. Despite having different background networks when investigating the newly appeared outflows, consistent outflow patterns could reveal that the newly appeared outflow is propagated from the network node close to the epileptogenic zone.

Due to the fact that the inter-ictal spikes and seizure discharging both result from neuronal abnormal discharging (Staley et al., 2005), our findings were similar with those of previous research (Burns et al., 2013), where they found that from the time of seizure onset to the middle or the end of seizure, the state of focus could switch from an isolated state to a connected state. Our results revealed that foci was actually changed from inactive to active approximately before discharging or during discharging. Based on the inter-ictal discharging EEGs, mainly within the time window of 460 ms, it has also been observed that in the left temporal lobe epilepsy group, the ipsilateral medial temporal pole serves as a key network hub node at the discharging moment (Coito et al., 2015). In our study for these three EPs, we found a similar pattern in that the network node close to the epileptogenic zone has important hub properties with large outflows. In another research based on Granger causality analysis the seizure was found to be propagated from medial temporal lobe to lateral temporal lobe, frontal lobe and so on (Jiao et al., 2009). Compared to the conventional Granger causality analysis, dynamic brain networks during inter-ictal discharging clearly revealed that the epileptogenic zone was activated prior to the onset of discharging and this worked as a source to propagate the activity to other brain regions. The time-varying network patterns specifically revealed by ADTF analysis were further utilized in the detection of the possible epileptogenic zone.

In this study we found that the propagation of brain activity from the local regions to the whole brain for all EPs. Moreover, the network node close to the epileptogenic zone is initially involved in the large outflows before the actual inter-ictal discharging and this may indicate the activation of epileptogenic zone to prepare for the incoming inter-ictal discharging. Therefore, it is worth noting that this phenomenon could serve as a biomarker for locating the epileptogenic zone. We established a procedure to automatically determine the epileptogenic zone based on the initial appearance of epileptogenic zone as the source node before actual inter-ictal discharging. The proposed approach determines F7, T4, and F3 as the possible epileptogenic zones for the three patients, which is close to clinical reports confirmed by epileptologists.

In conclusion, we employed the inter-ictal discharging data to construct the time-varying networks to account for the network mechanism of epilepsy and also proposed a method to localize epileptogenic zone based on a topological pattern of networks. Compared with previous studies based on the data of ictal period, the inter-ictal period data is much easier to be obtained clinically and interference from the other factors is weaker. In addition, in comparison with the postictal analysis, the inter-ictal network is more similar to the ictal network hence analysis based on inter-ictal period data may infer the neural mechanism close to that of the ictal period (Wilke et al., 2011).

A limitation of this research is that only three EPs were involved. In spite of the fact that preliminary findings can be revealed by this study, more EPs will be included in our future work to further verify the findings and the established approach for epileptogenic zone localization.

## Author contributions

LZ, FL, and PX conceived the data analysis procedure. LZ and YL performed the data analysis. LZ, FL, and PX wrote the paper. YL, HS, PD, and LY recorded the EEG data sets. WP, YS, and LS provided some useful suggestions in paper writing.

### Conflict of interest statement

The authors declare that the research was conducted in the absence of any commercial or financial relationships that could be construed as a potential conflict of interest.

## Appendix

### Pseudo-codes of our algorithm

Step 1. Calculating connectivity matrix *C* for each patient;

      Step 2. For *i* = 1 to *M*, (*M* represents the whole time point)

            For *j* = 1 to *N*, (*N* represents the number of electrodes)

                Out-degree matrix *O*(*i, j*) = sum (*C*(:, *j, i*) for electrode

                 *j* at time point *i*);

                 If *O*(*i, j*) < threshold *T* (i.e., *T* = 3) Then

                     Binarized out-degree matrix *BO*(*i, j*) = 0;

                 Else

                     Binarized out-degree matrix *BO*(*i, j*) = 1;

                 EndIf

              EndFor

           EndFor

      Step 3. For *i* = 2 to M

            For *j* = 1 to N

                 Difference *E* = *BO*(*i, j*) - *BO*(1, *j*);

                 If *E* == 1 Then

                     The brain area close to *j*th electrode is assumed as

                     the epileptogenic zone;

                         Stop;

                 EndIf

               EndFor

           EndFor
